# Clinical characterization of gingival type of burning mouth syndrome: a cross-sectional study

**DOI:** 10.4317/medoral.24791

**Published:** 2021-09-25

**Authors:** Noemi Coppola, Andrea Blasi, Massimo Amato, Roberto Ferrigno, Michele Davide Mignogna, Stefania Leuci

**Affiliations:** 1 Department of Neuroscience, Reproductive and Odontostomatological Sciences, Oral Medicine Unit, University of Naples Federico II, Naples, Italy; 2 Department of Medicine, Surgery and Dentistry “Scuola Medica salernitana”, University of Salerno, Salerno, Italy

## Abstract

**Background:**

The aim of this study is to investigate the prevalence of localized intraoral neuropathic pain in a cluster of patients who reported the involvement of gingival site as only clinical manifestation of dysesthesia, analysing type and distribution of symptoms.

**Material and Methods:**

Burning mouth syndrome (BMS) patients were enrolled in the study. Patients were screened through laboratory test and a conventional oral examination with periodontal chart. A questionnaire to collect data on symptoms, oral site involved, quality of sleep, anxiety was submitted to all the patients.

**Results:**

A total of 236 patients were recruited. Seventy-six patients (32.2%) presented generalized type, whereas 160 (67.8%) had localized type. In the localized BMS group, the gingiva was involved in 70 patients and in 33 of these it was the only site involved. In the gingival subgroup, 35 patients reported burning, 29 pain and 24 xerostomia.

**Conclusions:**

To best our knowledge, this study is the first that analyses gingival involvement as the only site in BMS and it could encourage further investigations to understand the etiopathogenesis of gingival BMS.

** Key words:**BMS, orofacial chronic pain, gingival BMS, localized BMS, localized neuropathic pain.

## Introduction

Orofacial pain (OFP) is a group of non-odontogenic disorders that encompass musculo-skeletal, neurovascular and neuropathic pain conditions affecting the head/neck area (1), which heterogeneous clinical manifestations are influenced by biological, psychological and social factors (1). Neuropathic pain is defined as “pain caused by a lesion or disease of the somatosensory nervous system” (2). Although the prevalence of neuropathic pain in the general population is unknown, chronic pain is one of the main reasons why patients seek medical attention (3). Like other types of chronic pain, also OFP is steadily increasing with approximately a quarter of the population reported at least once during the previous 6 months (1) and with a prevalence around 25% (4), representing a public health problem and interfering with quality of life (5). Neuropathic orofacial pain (n-OFP) is an underdiagnosed condition mostly by primary care where treatments represent a challenge for clinicians (6); the typical pattern followed by patients encompasses primarily the general dentist and then referred to oral medicine specialist or maxillo-facial surgeon (7). In consideration of the growing request for referrals, it is mandatory to update the knowledge on this field in order to provide a good level of medical assistance (4).

In chronic painful orofacial conditions, burning mouth syndrome (BMS) is included. BMS is an enigmatic pain condition in which the most common involved site is the tongue, especially anterior two-thirds and the apex, followed by the anterior palate and gingivae, the lower lips and the pharynx (8). It is necessary to know the prevalence of localized forms of BMS, in order to reduce the risk of misdiagnosis, especially those on which there is no data in the literature and the localized gingival neuropathic pain is one of them. OFP of periodontal origin can be caused by a wide range of diseases. The main causes of periodontal discomfort include inflammatory/reactive manifestations, infections and malignancies. In a recent review on periodontium as potential cause of OFP, the discomfort at gingival site is associated with acute infectious entities, herpetic lesions, periodontitis and necrotizing periodontal disorders, desquamative gingivitis, gingival recession, gingival enlargement and periodontal ligament strains (9). In addition, trigeminal neuralgia can be responsible for gingival pain causing electric shock-like pain over maxillary or mandibular gingiva (10). In the literature, no other neuropathic pain has been associated in detail with gingival discomfort.

The aim of this study is to investigate the prevalence of the neuropathic pain in gingival sites in BMS patients, analyzing type of symptoms and their distribution.

## Material and Methods

This observational and descriptive study was carried out at the Oral Medicine Unit, Federico II University of Naples, in a period between November 2019 and June 2020. The study protocol was approved by the local Ethics Committee (No. 125/19), conducted according to the guidelines of the World Medical Association Declaration of Helsinki and follows STROBE guidelines for the reporting of observational studies. BMS patients were consecutively enrolled during the first visit with an oral medicine specialist. Potentially eligible participants were identified on the basis of the following inclusion and exclusion criteria. The inclusion criteria were:

1. 18-70 years old patients of both genders

2. Patients with first diagnosis of BMS in accordance with the International Classification of OroFacial Pain (11)

3. Healthy oral mucosa

4. Negative laboratory findings (blood count, blood glucose, serum iron, ferritin and transferrin, folic acid and vit B12 levels).

The following exclusion criteria were applied:

1. Local or regional causes of symptoms

2. Systemic disorders, such as as hypertension, diabetes-mellitus, cerebrovascular diseases, neurodegenerative diseases, or laboratory abnormalities known to be potentially responsible for orofacial symptoms

3. Presence of periodontal inflammation (Bleeding on Probing-BOP≥30%).

Once included, patients’ general and dental history was investigated, and a conventional oral examination, including a periodontal chart, was carried out for all candidates to enrolment, in order to confirm BMS and exclude other synchronous oral diseases. Demographic and medical data of patients were collected, including data on anxiety and mood disorder diagnosed following the criteria of Diagnostic and Statistical Manual of Mental Disorders (DSM-V) during a psychiatric consultation, and a questionnaire created for the study was administered to all patients. The questionnaire investigated the following items: type of dysaesthetic sensation, oral site involved (tongue, soft palate, hard palate, cheek, lip, gingiva, teeth), quality of sleep, free comment section.

- Statistical Analysis

The following parameters were recorded for each patient: age, gender, smoking habits, alcohol assumption, duration of recurrent dysesthesia, presence of systemic conditions, presence of mood disorders, symptoms and extension of BMS (localized if less than three sites are involved or generalized if more than three sites are involved), affected sites.

Age and duration of recurrent dysesthesia were reported as mean and standard deviation. Gender, smoking habits, alcohol assumption, presence of systemic conditions, presence of mood disorders, extension of BMS were dichotomous variables and reported as percentages. Systemic conditions, mood disorders, BMS symptoms and sites were summarized as categorical variables and reported as percentages. Smoking level, a quantitative data, was categorized into 3 groups: less than 5 cigarettes per day, between 5 and 15 cigarettes per day, and more than 15 cigarettes per day.

A comparison between localized and generalized forms of BMS was carried out with chi-square test in order to assess differences in terms of age, gender, smoking habits, alcohol assumption, presence of systemic conditions and presence of mood disorders.

Dedicated software (IBM SPSS Statistics for Windows, Version 25.0. Armonk, NY: IBM Corp.) was used for all statistical analyses.

## Results

A total of 238 patients were recruited, 236 of which agreed to participate to the study (99.2% participation rate) and successfully completed the questionnaire autonomously. The mean age of the patients was 58,5±13,5 years and the sample was characterized by a significantly higher rate of female gender. The prevalence of high exposure level to risk factors such as smoking and alcohol use was significantly higher in male patients (*p*<0.0001) (Table 1).

**Table 1:**
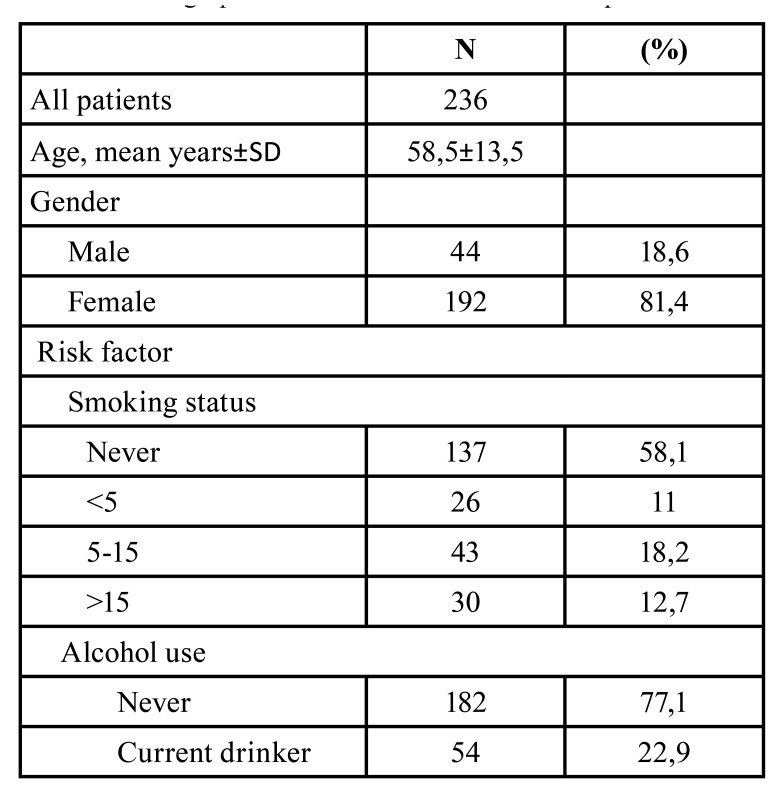
Demographic characteristics of the enrolled patients.

On the basis of number and type of affected sites we identified three forms of BMS: generalized (more than three sites involved), localized BMS without gingival involvement (BMS-G) (less than three sites involved) and localized BMS with gingival involvement (BMS+G)

The three groups did not show significant differences in age, education, gender, and employment status.

In the total sample the most common involved site was the tongue (150, 63.6%) followed by gingiva (146, 61.9%) and palate (107, 45.3%). In the total of enrolled sample, the most prevalent symptom was burning (104, 44,8%), followed by xerostomia (95, 40,3%) and pain (60, 25.4%).

Seventy-six patients (32.2%) presented generalized type, whereas 160 (67.8%) had localized type with a similar distribution (tongue 74, 46.3%- gingiva 70, 43.8%-palate 31, 19.4%).

Where gingival dysaesthetic sensation was reported, the upper gingiva was involved in 57 cases (81.4%), the lower gingiva in 49 cases (70%) and the retromolar triangle in 6 cases (8.6%). In 33 (47.1%) patients the gingiva was the only site involved while in 37 (52.9%) patients gingival involvement was associated with other sites with the following prevalence: tongue in 15 (40.5%) patients, palate in 11 (29.7%), teeth in 9 (24.3%) and cheek in 7 (18.9%).

In patients with generalized form of BMS, the most common symptom was burning (37, 48.7%), followed by xerostomia (33, 43.4%) and subjective sialorrhea (20, 26.3%); in the gingival group, 35 (50%) patients referred burning, 29 (41.4%) pain, 24 (34.3%) xerostomia. The prevalence of oral symptoms in each subgroup is described in Table 2.

**Table 2:**
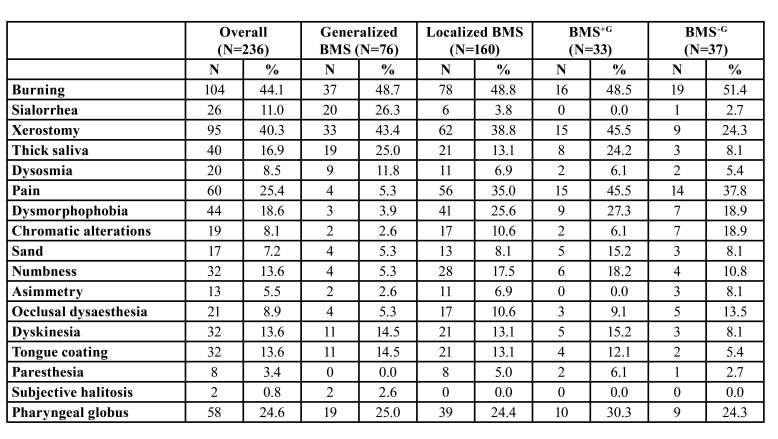
Prevalence of oral symptoms in each subgroup.

Globus was present in 25% of generalized BMS group, in 22,2% of localized BMS-G and in 27.1% of BMS+G.

Sleep disturbances have been reported by 53.9% patients with generalized BMS, 65.6% with localized BMS-G and 51.4% with BMS+G.

As regards to anxiety and depressive syndrome, in the generalized BMS group 56.6% and 25% of patients were diagnosed with anxiety and depression respectively, in localized BMS-G group 63.3% and 22.2%, while in the BMS+G group these diagnoses were present in 44.3% and 14.3% of patients. The difference between the BMS+G and BMS-G was statistically significant for state anxiety (*p*= 0.012).

## Discussion

BMS is a chronic painful condition classified according to the International Classification of Orofacial Pain as a painful cranial neuropathy characterized by an intraoral burning sensation or dysesthesia, recurring every day for more than 2 hours/day for more than 3 months, without clinically evident causal lesions (11). The prevalence is 3.7% in adult population mostly in postmenopausal women (12). The aetiology is still poorly understood and several mechanisms are involved in its onset including central and peripheral nerve alterations and psychological factors (13). Neuropathic alterations at different levels of the nervous system in BMS have been documented. Previous studies have demonstrated changes in the medial system of the pain-related network (14), structural and functional deficits in the medial prefrontal cortex and hippocampus (15) and changes in grey matter structure and white matter structure (16). Also peripheral nervous alterations, such small fibres neuropathies, are involved in BMS onset (17); in particular, involvement of small myelinated A and unmyelinated C fibres has been demonstrated (17).

The most common involved site is the tongue (8), while the most common reported symptoms are: burning, xerostomia, itching, stinging, pain, sand sensation, dysgeusia and paresthesia (8).

In line with the literature, in our sample the predominant involved site was the tongue and the main symptom why patients request for visit was burning. Although site and symptoms are consistent with reported data, it is interesting to note the high number of patients who reported localized involvement. In fact, in our cohort 160 patients were diagnosed with localized BMS and 70 of them had gingival involvement. Thirty-three patients reported symptoms exclusively in the gingival area; in cases where the dysaesthetic symptoms were also localized in other sites besides the gingival one, the main site was the tongue, confirming to be the most involved site, followed by palate and teeth. Also in BMS+G patients the main symptom was burning confirming that it is the characteristic BMS symptom. While in the generalized BMS group the second most frequent symptom was xerostomia, in the gingival BMS group it was pain, expression of a localized superficial sensory alteration.

Anxiety, depression and sleep disturbances are often BMS comorbidities (18) and the overlap with other chronic painful disorders is widely documented (19-20). In our sample 50-65% of patients report sleep disorders with no statistically significant differences between the three compared groups. We confirmed published data about the association between BMS and anxious and depressive syndrome with no statistically significant differences between the three groups examined, except for the anxiety which is statistically significantly lower in patients with gingival BMS than in other localized forms of BMS. The absence of significant differences in the three groups for sleep disturbance and mood suggests that the onset of sleep and psychological disorders is not linked to peripheral but to central neuropathy in the course of the disease.

The heterogeneity of the reported symptoms and the multiplicity of the involved sites make difficult a proper diagnosis that still represent a challenge for clinicians; the co-occurrence of BMS, poor sleep, anxiety and depression increases the burden of the disease and make therapy more complex. As dentists are often the first clinicians to visit patients affected by OFP and BMS (21), they need to be able to recognize common forms of BMS and its variants, such as gingival localization. Adequate knowledge of non-dental orofacial pain among general dentists is necessary in order to avoid inappropriate dental treatments and provide a correct diagnosis and treatment to the patient. The data from several studies demonstrate a lack of knowledge and attitude about OFP in dental practice (22-23); when the patient reports pain with no evidence of odontogenic cause, often the general dentist practitioners don’t hypothesize a non-dental origin initiating dental treatments anyway rather than referring patient to the specialist (24). This attitude can lead to misdiagnosis and inappropriate treatments, especially when the neuropathy involves sites that are easily and frequently affected by infections and inflammation, such as gingiva, and when this area is the only one involved. In our cohort, 33 patients reported dysesthesia localized exclusively in the gingival district, representing a non-negligible percentage of the sample. In these patients, periodontal inflammation or other causes that could determine periodontal discomfort, such as dental materials in contiguity with periodontal soft tissues, were not identified. Therefore, if conventional oral examination and periodontal examination exclude local causes of periodontal discomfort, the hypothesis of neuropathic pain must be considered. Pain in the gingival tissue is a superficial somatic pain (25). Several studies demonstrated the presence of Aδ and C fibres in the periodontal ligament that playing a crucial role in nociception (26). Nociceptive pain is defined as pain triggered by a noxious stimulus, in contrast to neuropathic pain that is caused by a lesion or disease of neural tissue (27). In fact, neuropathic pain affects the central and peripheral nervous systems and the related dysfunctions represent the main hypothesized pathogenetic mechanism of BMS (28). Although neuropathic pathogenesis has been widely demonstrated, there are no data on the pathogenesis of the localized forms of this pathology. Recent work based on chronic pain demonstrated that some chronic pain conditions have nociceptive and neuropathic components (29) and the term “mixed pain” was used, such as for low back pain, although it has not yet been formally defined (30). In consideration of the lack of knowledge about localized type of BMS, it is interesting analyse whether the peripheral nociceptive component may play a more important role than the generalized form in onset of localized BMS.

To best our knowledge, this study is the first that analyses localized gingival involvement in BMS patients and it could encourage further investigations to understand the etiopathogenesis of this form of BMS.
